# Pediatric hanging injury: Rapid intervention and full neurological recovery

**DOI:** 10.1016/j.radcr.2025.06.007

**Published:** 2025-06-28

**Authors:** Matyas Wondwossen Elssa, Merahi Kefyalew Merahi, Kebron Wossen Aweke, Bement Girma Abera, Kidist Nega Aragaw, Kefelegn Negalign Mekuria, Daniel Berhane Gebresilassie

**Affiliations:** a College of Health Science, School of Medicine, Addis Ababa University, Addis Ababa, Ethiopia; b College of Health Science, School of Medicine, Wolkite University, Wolkite, Ethiopia; c College of Health Science and Medical Sciences, Dilla University, Dilla, Ethiopia

**Keywords:** Pediatric hanging, Hypoxic encephalopathy, Suicide attempt, Multidisciplinary care

## Abstract

Hanging injuries are a subset of strangulation injuries that pose significant risks of hypoxia and neurological damage, especially in pediatric populations. This report presents a case of a 13-year-old boy with hypoxic brain injury following a suicide attempt by hanging. Key aspects include the prolonged duration of strangulation, absence of vertebral or laryngeal trauma, successful intensive care management, and full recovery without residual neurological deficits. This case emphasizes the importance of timely intervention, multidisciplinary management, and preventive education to address the acute and long-term challenges of pediatric hanging injuries.

## Introduction

Hanging is defined as the suspension of a person by a noose or ligature around the neck. Strangulation, in contrast, is the constriction of a body part—specifically, in the case of the neck, it involves suffocation through compression of the trachea or upper airways. Thus, hanging constitutes a subcategory of strangulation [1].

Strangulation injuries represent critical emergencies requiring immediate intervention due to the significant risk of hypoxia and neurological injury [2]. We describe the case of a 13-year-old patient who sustained hypoxic brain injury following a suicide attempt by hanging. This case highlights the prolonged duration of strangulation, the absence of vertebral or laryngeal trauma, the critical importance of early intervention, successful PICU management, and the need for preventive education.

## Case presentation

A 13-year-old male was found suspended by a cotton belt for approximately 13 minutes. His tutor discovered him and immediately lowered him. At the time of discovery, he was unconscious with audible breathing. He was transported to a local health center, where he received oxygen support and was urgently referred to Tikur Anbesa Specialized Hospital for further evaluation and management.

Upon arrival in the emergency department, the patient had a Glasgow Coma Scale (GCS) score of 8/15, indicating significantly altered consciousness. Initial vital signs were: pulse rate 131 bpm, blood pressure 112/70 mmHg, respiratory rate 40 breaths per minute, and oxygen saturation 90% on a face mask.

## Primary survey

*Airway:* The airway was not patent or protected. Audible stridor and secretions were present. Rapid sequence intubation (RSI) was performed using intravenous propofol (1 mg/kg), succinylcholine (1 mg/kg), and diazepam (0.1 mg/kg). A 5.5 mm endotracheal tube was successfully placed on the first attempt with in-line cervical spine stabilization.

*Breathing:* The patient was placed on mechanical ventilation (VAC mode) with FiO₂ 100%, tidal volume (TV) 240 mL, respiratory rate 16 breaths/min, and PEEP 5 cm H₂O. Oxygen saturation improved to 100%. Spontaneous respiratory effort was noted with a rate of 28 breaths/min.

*Circulation:* Blood pressure was 114/60 mmHg and pulse rate 110 bpm. Extremities were warm with brisk capillary refill. No active bleeding was noted, and the pelvis was stable.

*Disability:* GCS remained 8/15 (E1, V2, M5). Pupils were mid-sized and bilaterally reactive. The patient moved all extremities spontaneously.

**Fig. 1:**
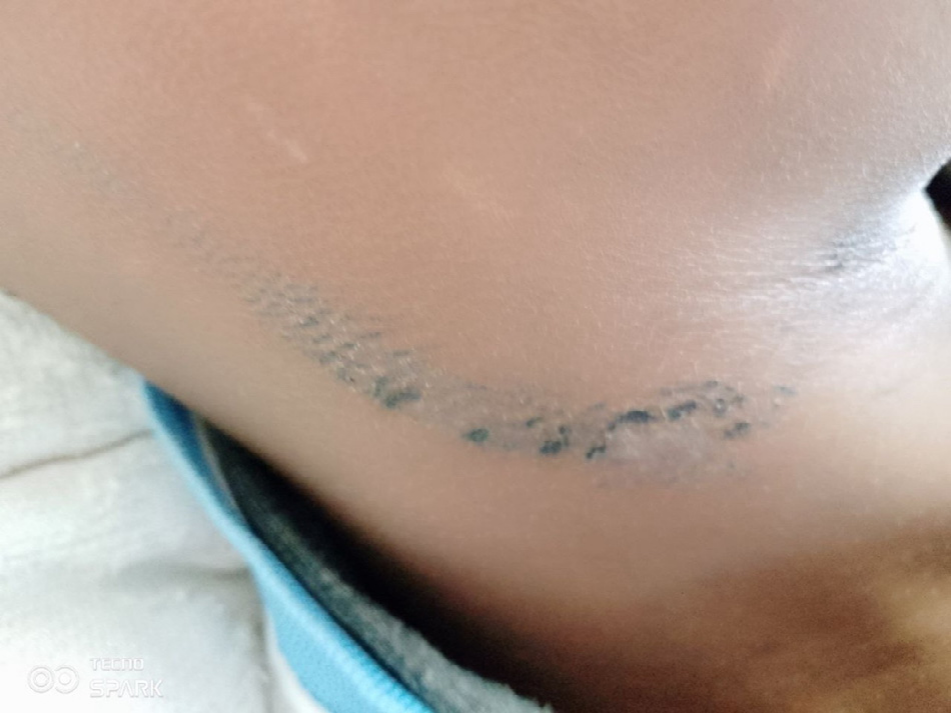
Bruising from the incident on patient’s neck.

*Exposure:* No active external bleeding was observed. A rope bruise was present on the neck (Fig. 1). Focused Assessment with Sonography for Trauma (FAST) and extended FAST (E-FAST) examinations were negative.

Following stabilization, secondary survey and comprehensive physical examination revealed no significant past medical history or signs of underlying illness. The patient had no prior psychiatric diagnoses or documented depressive symptoms. However, poststabilization psychosocial evaluation identified significant stressors. He reported intense academic pressure related to his high school performance ranking and described social adjustment difficulties after transferring to a new school 2 years earlier. Although he eventually formed friendships, his mother noted recent behavioral changes, including school tardiness and frustration over repeatedly losing his ID card. These stressors culminated in him verbalizing, “I will kill myself,” to peers prior to the event. His mother expressed surprise at his actions, stating he appeared calm and communicative following resuscitation, without overt fear or remorse. Mental status examination revealed a cooperative, seemingly overly friendly demeanor, normal speech, appropriate eye contact, and a hyperpigmented neck ulcer secondary to the ligature.

**Fig. 2:**
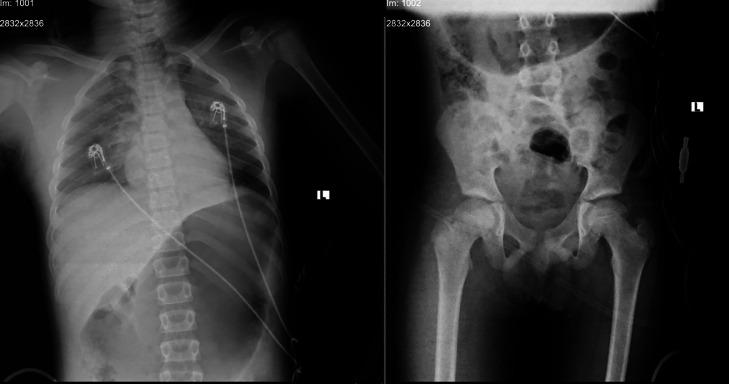
Chest and pelvic x-ray of the patient.

**Fig. 3:**
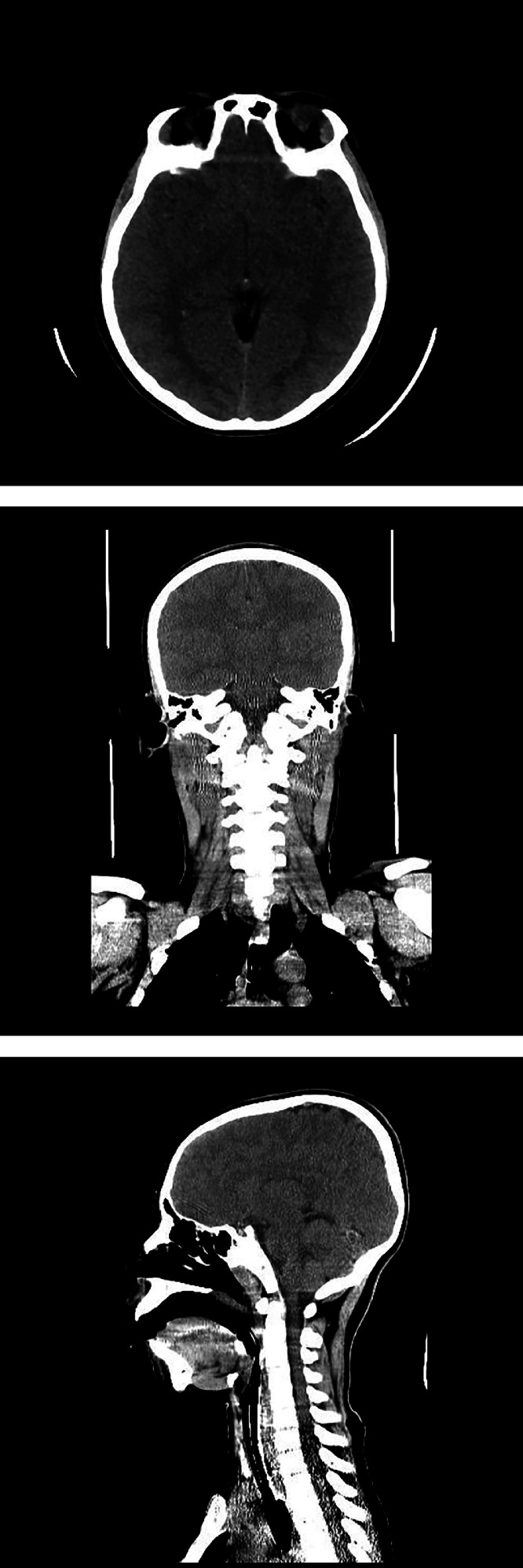
Noncontrast brain CT of the patient.

*Assessment and Management in the Pediatric Intensive Care Unit (PICU):* The immediate assessment indicated hypoxic encephalopathy secondary to a strangulation injury due to a probable suicide attempt. He was monitored while receiving mechanical ventilation. Baseline laboratory investigations were performed, including complete blood count (CBC), serum electrolytes, and organ function tests. A noncontrast brain CT scan and a chest and pelvic X-ray were obtained (Fig. 2). Noncontrast brain CT revealed no acute intracranial pathology. Normal grey-white matter differentiation was preserved throughout all visualized brain regions. There was no evidence of cerebral edema, mass effect, or hypoxic-ischemic injury within the sensitivity limits of CT (Fig. 3).

Management included the administration of maintenance intravenous fluids, sedatives, and analgesics (morphine, ketofol, fentanyl). Additional therapies comprised omeprazole for stress ulcer prophylaxis, dexamethasone for cerebral edema, and regular nebulization.

## Outcome

The patient was admitted to the Pediatric Intensive Care Unit (PICU) for 15 days, demonstrating significant clinical improvement throughout his stay. He was successfully weaned from mechanical ventilation and extubated. Following rigorous physiotherapy, he was discharged without residual neurological deficits. A psychiatry consultation was obtained, and he was referred to the outpatient department for scheduled follow-up after an initial inpatient evaluation.

## Discussion

Suicide attempts among children under 14 years, particularly males, are relatively uncommon but increasingly reported, partially attributable to mental health disorders and environmental stressors [3]. Self-strangulation represents the most frequent suicide method in this demographic [4]. Strangulation injuries encompass diverse complications including hypoxia, airway edema, and neurological damage secondary to ischemia. The pathophysiology of hanging involves airway obstruction and venous congestion, culminating in hypoxic-ischemic injury that clinically manifests as seizures or altered consciousness [5,6].

Pediatric patients face substantially higher risks of airway edema compared to adults due to anatomical differences in respiratory structures. While greater tissue elasticity reduces susceptibility to laryngeal or vertebral fractures following strangulation [1], children exhibit heightened vulnerability to airway compromise and respiratory distress [7,8]. This risk escalates significantly with prolonged strangulation duration.

The absence of abnormalities on noncontrast brain CT, such as loss of grey-white matter differentiation or cerebral edema, aligns with literature indicating that early imaging may not detect transient hypoxic insults. While MRI with diffusion-weighted imaging (DWI) and apparent diffusion coefficient (ADC) sequences is superior for identifying acute ischemic changes, this modality was not pursued due to limited availability of MRI resources at the institution. This case emphasizes the importance of clinical correlation, as imaging findings may lag behind functional recovery, particularly in children with greater neuroplasticity.

Optimal management of strangulation injuries necessitates multidisciplinary collaboration among emergency physicians, intensivists, and mental health professionals [2]. Immediate airway control through rapid intubation and mechanical ventilation is critical to prevent hypoxic deterioration [9]. Appropriate sedation and analgesia are essential for postintubation care, while vigilant monitoring for complications such as ventilator-associated pneumonia remains imperative during prolonged ventilation [7,8]. In this case, prompt airway management and intensive care significantly contributed to the patient's rapid recovery despite profound initial hypoxia.

Psychological implications must be systematically addressed. Although this patient denied prior suicidal ideation, significant psychosocial stressors emerged—including academic pressure, social isolation following school transition, and frustration over recurrent ID losses. His explicit verbalization of suicidal intent to peers, coupled with observed behavioral changes (tardiness, emotional distress), highlights how undetected distress may precipitate pediatric suicide attempts. This case emphasizes the necessity for routine psychosocial screening in adolescents, even absent formal psychiatric diagnoses, particularly given evidence that mental health disorders frequently remain undetected in preadolescents [7,10]. Since many pediatric strangulation attempts correlate with underlying psychological or environmental factors [6,7,11], comprehensive psychiatric assessment is vital for both recovery and prevention.

Prevention strategies should extend beyond acute care to include: school-based mental health initiatives (eg, stress management workshops, peer support programs); caregiver education on recognizing subtle behavioral changes; and public health campaigns targeting suicide prevention during academic or social transitions.

In this case, psychiatric evaluation was initiated following medical stabilization. The patient reported no recollection of the incident and denied current depressive symptoms or suicidal ideation during initial assessment. Nonetheless, ongoing outpatient supportive psychotherapy was implemented. This ongoing care remains crucial, as longitudinal follow-up allows for monitoring emotional adjustment, identifying emerging mental health needs, and reinforcing adaptive coping strategies.

## Conclusion

This case illustrates the distinct clinical and anatomical considerations of pediatric hanging injuries. It underscores the critical importance of rapid intervention and a multidisciplinary approach to management. Clinicians must maintain a high index of suspicion for subtle presentations to optimize neurological outcomes. Finally, integrated prevention strategies—including caregiver education, school-based mental health support, and public awareness initiatives—are essential for mitigating such incidents in vulnerable pediatric populations.

## Patient consent

We confirm that written informed consent was obtained from the patient's legal guardian for the publication of this case report and any accompanying images. The patient's anonymity has been preserved, and no identifying information has been disclosed. According to our institution's policies, ethical approval was not required for this case report.

## CRediT authorship contribution statement

**Matyas Wondwossen Elssa:** Conceptualization, Writing – original draft, Visualization. **Merahi Kefyalew Merahi:** Resources, Supervision. **Kebron Wossen Aweke:** Resources, Writing – original draft. **Bement Girma Abera:** Writing – review & editing. **Kidist Nega Aragaw:** Writing – review & editing. **Kefelegn Negalign Mekuria:** Writing – review & editing. **Daniel Berhane Gebresilassie:** Resources.
